# Potential therapeutics ingredients for dental pulp regeneration focusing on DPSCs and DPSC secretome with market projection

**DOI:** 10.1177/09636897251414002

**Published:** 2026-09-28

**Authors:** Vinita Dsouza, Amrutha Hebbar, Shashi Rashmi Acharya, Raviraja N. Seetharam, Kirthanashri S. Vasanthan

**Affiliations:** 1 Department of Biotherapeutics Research, Manipal Academy of Higher Education, Manipal, India; 2 Manipal College of Dental Sciences, Manipal Academy of Higher Education, Manipal, India; 3 Chandigarh University, Mohali, India

**Keywords:** dental pulp stem cells, DPSC secretome, regenerative endodontics, three-dimensional scaffolds, pulp–dentin complex regeneration, paracrine signalling

## Abstract

Regeneration of dental pulp has surfaced as an exciting area of exploration in regenerative endodontics, as a result of progress in stem cell biology and their applications, along with biomaterials. Of the different cell populations studied, dental pulp stem cells (DPSCs) are recognized as possessing significant regenerative potential considering their multipotency, immunomodulatory properties and capacity to differentiate into odontoblast-like cells. Interest has also increased in alternative approaches, such as the DPSC-derived secretome, which serves as a bioactive consulting of molecules like cytokines, growth factors and extracellular vesicles, to initiate regenerative processes through paracrine signalling without the limitations of cell-based treatments. This review highlights the relative therapeutic potential of DPSCs secretome when integrated with three-dimensional scaffolds for the regeneration of the pulp–dentin complex. We discuss the biological mechanisms by which DPSCs secretome helps in regeneration assess the compatibility of scaffolds, and review advantages and limitations regarding their use based on efficacy, immunogenicity, storage and clinical use. Although DPSC scaffolds provide direct tissue reconstruction, secretome-enhanced scaffolds are clinically applicable as a cell-free, scalable and potentially safer method. A better understanding of the advantages and disadvantages of DPSC secretome-modified scaffolds will assist in the development of future and emerging translational opportunities for personalized, minimally invasive regenerative endodontic therapies.

## Graphical abstract

**Graphical Abstract:**
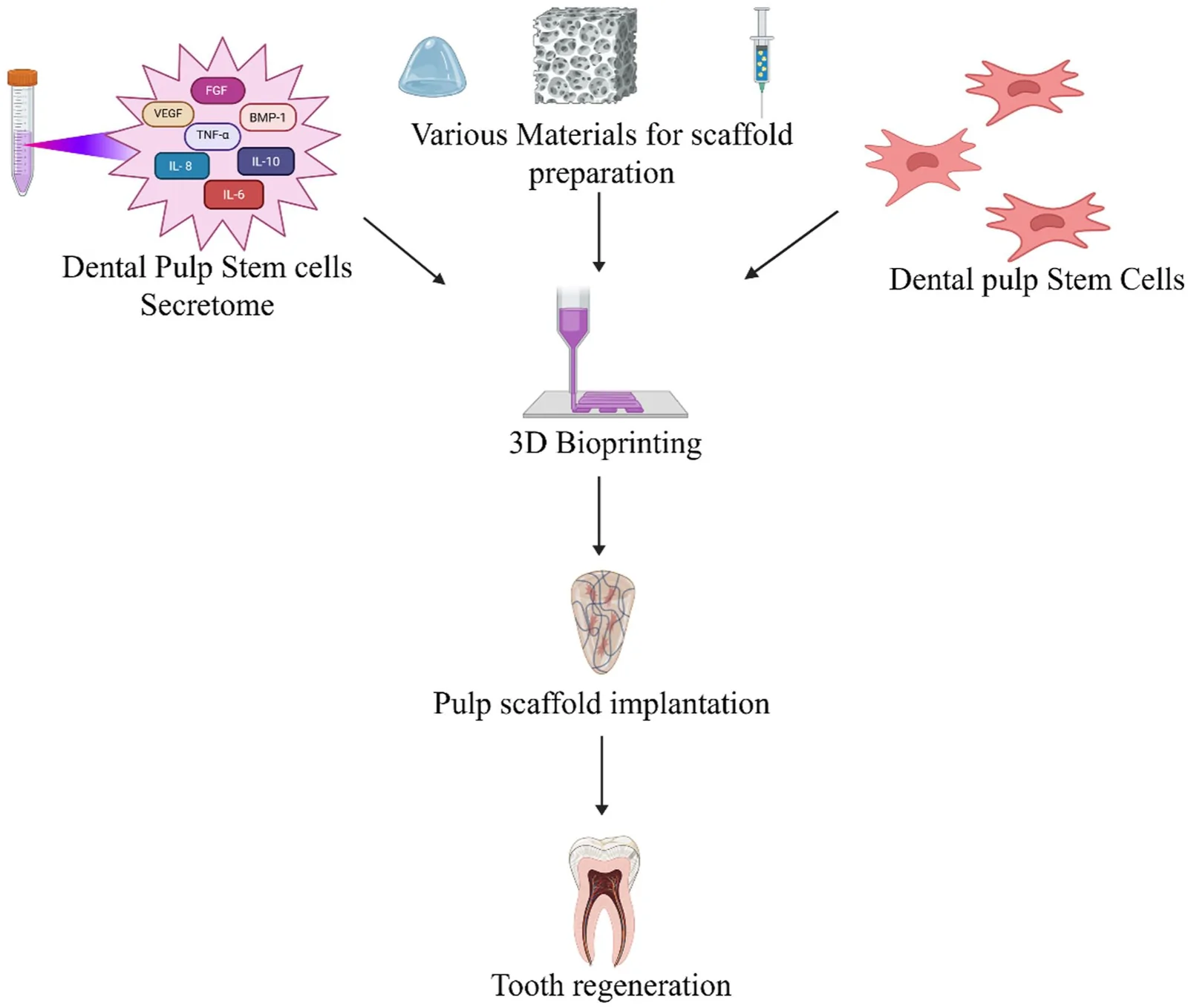
Overview of Pulp regeneration (Figure created by the authors using BioRender.com)

## Introduction

Dental pulp vitality is very important to maintain the physiological function and tooth integrity. There are various treatments such as the root canal therapy and extraction and regenerative endodontic procedures these intend to restore the pulp–dentin complex these treatments were successful in eradicating infection, but they would make the tooth non-vital and prone to structural weakness^1,2^. The area of regenerative endodontics has developed an aim to restore the pulp–dentin complex biological and functional characteristics^2,3^. Restoring the complete functionality of necrotic teeth with immature roots is an intriguing objective since patients’ quality of life is enhanced by the regeneration of lost tissues^3,4^. Dental implants are used in various treatments as they offer a functional and aesthetic alternative to natural teeth. Most implants are made of biocompatible materials and are usually made of titanium or zirconia, and they support dental prostheses like crowns, bridges or dentures. While implants are inserted in the jawbone surgically, they become integrated in such a way that they fuse with the bone through a process known as osteointegration. Dental implants have experienced considerable advancements in materials, designs and approaches since their introduction in the mid-20th century, improving durability, functionality and patient outcomes^5^. However, some challenges include lengthy healing time or risk of infections, as well as biocompatibility challenges, which can slow or complicate the recovery process for the patients^6^. To overcome these issues, researchers are exploring alternative approaches through innovative technologies, such as 3D printing and current bioprinting technologies^3^. The development of tissue engineering-based regenerative dental pulp therapies is a practical application of ongoing scientific advancements. There has been a steady advance in the field of stem cell biology, offering treatments for missing or damaged tooth tissues. The field of 3D printing dental implants has progressed rapidly, and additive manufacturing opens new opportunities for manufacturing complex geometries and patient-specific implants that can achieve unrivalled accuracy^7^. This method of 3D printing implements computer-assisted design (CAD) and builds these structures layer by layer. Using this approach allows for the customization of a patient-specific implant based on the patient’s anatomy. These constructions had more favourable surface qualities that assisted controlled surface textures promote osseointegration via controlled surface textures^8^. It has also been shown to have a reduced lead time compared to traditional methods of manufacturing^9^. Recent literature looks at the potential of materials such as titanium alloys, ceramic composites and polymers for use in 3D-printed implants^10^. For instance, titanium specifically made through selective laser melting (SLM) demonstrated better mechanical properties and biocompatibility^7^. Bioprinting improves on standard 3D printing by integrating bioactive materials and living cells into the manufacturing processes. This manufacturing method seeks to develop an implant that not only replaces lost structures but also establishes eventual biological integration with host tissues and promotes tissue regeneration^8^. These include scaffolds made from hydrogels that would be seeded with stem cells known to enhance growth in both bone and soft tissues^11^. Bioprinted implants also have the potential to release bioactive molecules, such as BMP-2, to optimize bone formation. Additionally, both hard and soft materials can be manufactured with the aim of mimicking the natural interface between bone and soft tissues^9^. A publication in Biomaterials Science has outlined the advantages of using lattice structures in titanium implants for weight reduction and the enhancement of osseointegration^8^. The use of 3D-printed lattice structures displayed superior mechanical stability, and bone-integration characteristics were investigated (Table 1). Current research is now examining the use of 3D-printed zirconia for patients with metal allergies or aesthetic concerns, which offers high strength and colour that resembles a natural tooth while meeting the modern demands of dentistry. Studies have successfully combined dental pulp stem cells (DPSCs) with bioprinted scaffolds for root regeneration^10–13^. These scaffolds not only support the growth of cells but also promote healing time while minimizing complications. Innovations have included coating implants with bioactive materials (e.g. silver nanoparticles) to decrease bacterial colonization and prevent peri-implantitis^12–15^. Recent research has shown that coating has also been integrated during the 3D printing process^16^. Bioprinting techniques have also created scaffolds preloaded with either osteoblasts or mesenchymal stem cells, formulated to help repair the severe alveolar bone loss and provide support for future implants. The practice of dentistry is advancing with 3D printing and bioprinting technologies that presented claims of accurate and physiologically useful solutions. Future directions encompass fully bioprinted teeth, along with the optimization of materials that would improve the likelihood of long-term success, as well as AI-infused customizations that would lead to improved outcomes for patients. With these developments, technology is on the verge of transforming dental care with respect to restorative and regenerative therapies. Working through limitations and supporting individualized patient needs, promising improvement of oral health and quality of life can be made possible through dental implants that are either 3D-printed or bioprinted. There are two general translational paths that are currently being explored such as cell-based therapies and cell-free strategies; these use the bioactive secretions from stem cells rather than live cells. The dental pulp is regarded as a potential source of mesenchymal stem cells (MSCs) for protocols because the method of collecting the MSCs is not an invasive one and can be facilitated by a routine procedure, with respect to wisdom teeth^13,16,17^. Therefore, tissue loss can essentially be avoided. Due to their unique properties, dental pulp stem cells (DPSCs) have recently risen to a prominent position in the area of regenerative medicine^18^. Dental pulp stem cells are a particular type of mesenchymal stem cell derived from dental pulp. These stem cells can be characterized by their high plasticity, significant proliferative potential and ability to differentiate into a variety of cell types, osteoblasts, chondrocytes and possibly neurons^19^. DPSCs innate characteristics allow them to serve as a beneficial cell type for regenerative medicine in general, particularly tooth pulp repair and regeneration. DPSCs’ regenerative ability is mainly attributed to their secretome, which consists of many bioactive substances, released by DPSCs to help cell–cell interactions and repair^18,19^. DPSCs were recently discovered (2007), thus providing an even greater understanding of another form of adult stem cell with mesenchymal attributes that may be readily acquired from teeth after extraction for regenerative therapies. DPSCs also have several distinguishing characteristics, including the ability to self-renew, differentiate into various lineages and secrete a wide variety of growth factors and cytokines that facilitate healing and regeneration^20^. The significance of DPSCs reaches beyond the field of dentistry^21–24^. To date, secretome studies have determined regenerative properties in connection with a variety of ailments and disorders, such as neurological disease models and disorders relating to cartilage. The secretome, which encompasses extracellular vesicles (EVs), soluble factors and proteins, working paracrine action, can mediate various effects and amplification of regeneration for the tissue surrounding them^25,26^.

**Table 1.** Various scaffolds with DPSC used in different applications.

Type of bioprinted dental implant	Dental stem cells used	Description	References
Titanium-lattice implants	Dental pulp stem cells (DPSCs)	3D-printed titanium implants with lattice structures designed to improve osseointegration. DPSCs enhance bone regeneration and tissue integration.	Sushmita et al.^27^; Wang et al.^28^; and Li et al.^29^
Zirconia implants	Dental pulp stem cells (DPSCs)	Bioprinted zirconia implants offering high strength and aesthetic benefits, particularly for patients with metal allergies. DPSCs may be incorporated for tissue regeneration.	Sui et al.^30^
Hydrogel-based bone regeneration scaffolds	Mesenchymal stem cells (MSCs), DPSCs	3D-printed hydrogel scaffolds infused with DPSCs or MSCs to stimulate bone growth and support bone regeneration in implant sites.	Xie et al.^31^ and Kim et al.^32^
Bioactive coated implants	Dental pulp stem cells (DPSCs), stem cells from apical papilla (SCAP)	Implants coated with bioactive materials like silver nanoparticles, with DPSCs or SCAP promoting healing and preventing infection at the site of implantation.	Zhang et al.^33^ and Kim et al.^34^
Regenerative root canal implants	DPSCs, SCAP, periodontal ligament stem cells (PDLSCs)	Bioprinted scaffolds for root canal regeneration, utilizing DPSCs, SCAP and PDLSCs to regenerate damaged pulp tissue and facilitate osseointegration.	Lee et al.^35^
Multi-material implants	DPSCs, MSCs, oral epithelial stem cells	Bioprinted implants combining hard and soft tissue materials with DPSCs and MSCs to support both bone and gingival regeneration.	Park et al.^36^ and Wu et al.^37^
Personalized bioprinted implants	Dental stem cells from exfoliated teeth (SHED)	Customized implants based on 3D scans of the patient’s oral cavity, utilizing SHED cells to regenerate teeth and support bone healing.	Lee et al.^38^

Dental pulp stem cells (DPSCs) are a new and promising type of stem cell that have generated considerable interest in the field of regeneration medicine and tissue engineering^19,39^. The discovery and characterization of these cells have created possibilities for further therapeutic opportunities, especially in the area of dental and craniofacial regeneration^11,40^. This review describes the background of DPSCs and its secretome that will encompass their origin, properties, isolation methods, differentiation potential and clinical relevance.

## Biology of DPSCs and their secretome

### Origin and discovery of DPSCs

In 2000, Gronthos et al.^20^ were the first to isolate and characterize DPSCs from human impacted third molars. This work established that these cells exist in the dental pulp, which is the soft tissue that contains nerves, blood vessels and connective tissue inside the teeth. The discovery of DPSCs represented a significant milestone in stem cell research as they were one of the first adult stem cells discovered in the orofacial area^20,41^. The embryonic origin of DPSCs is as neural crest cells, which contribute to the formation of multiple tissues including dental tissues^42^. The multipotency of DPSCs is a result of their neural crest derivation and ability to differentiate into multiple lineages (e.g. neurons, adipocytes, chondrocytes and osteoblasts) under appropriate conditions^21,43,44^. DPSCs have several defining characteristics that differentiate them from other stem cell populations, for example, DPSCs exhibit pluripotency, where they form embryoid body-like structures *in vitro* and teratoma-like structures when injected into immunocompromised mice, demonstrating the capability to give rise to cell types from all three embryonic germ layers^45,46^. Data concerning dental pulp stem cells (DPSCs) shows that they have around a 30% greater rate of proliferation than other mesenchymal stem cell (MSC) types like bone marrow stromal stem cells (BMSSCs)^47–49^. This more significant proliferation is associated with elevated levels of cell cycle regulators such as cyclin-dependent kinase 6 (CDK6) found in dental pulp^50^. DPSCs also show reduced immunogenicity compared to other MSCs, meaning they are less likely to initiate an immune response post-transplantation^51^. Longitudinal investigations revealed that DPSCs are not a homogeneous population, with various subpopulations defined by distinct phenotypic traits^52,53^. This variation may have an impact on their therapeutic and regenerative capabilities.

### Different methods of DPSC cell extraction

Dental mesenchymal stem cells are multipotent adult mesenchymal stem cells originating from ecto-mesenchymal neural crest cells. In recent years, these cells have gained a lot of traction in the field of research^20,41^. There are various types of dental mesenchymal stem cells isolated from various parts of the oral cavity such as SHEDs isolated from the deciduous tooth and DPSCs isolated from the pulp of the third molars^54–56^ (Fig. 1). The isolation of these cells would be done by using either primary explant culture or enzymatic dissociation methods^57^. In the primary explant culture, the isolated tooth pulp would be cut into pieces and plated onto the culture dish with media in it and observed for cells to grow from the explant^57,58^. In case of enzymatic method, the pulp tissue would be treated with collagenase and dispase and then centrifuged, the pellet is resuspended in media and then its seeded into the culture flask and observed for the cell growth^57,58^. Gronthos et al.^20^ was the first to discover the dental pulp stem cells; they used the enzymatic dissociation method to obtain the cells and proved that these have the potential to form odontoblasts, and their study also proved that these cells were capable of forming the dentin–pulp matrix *in vivo*. Similarly, Miura et al. used a combination of collagenase and dispase to isolate DPSCs from extracted human teeth. The isolated cells were expanded in culture and demonstrated the ability to differentiate into odontoblast-like cells, further confirming their stem cell properties^53,59^. This study also employed collagenase digestion to obtain DPSCs, which were then cultured in α-MEM with 20% FBS. The DPSCs proved they could differentiate into multiple types of cells, including odontoblasts and adipocytes, and showed they were multipotent. Laino et al. used the explant culture method to obtain the DPSCs from the dental pulp of human third molars. This involved dividing the pulp tissue into small pieces and putting them in culture plates with DMEM with 20% FBS^60^. The cells that grow out from the explants were collected and propagated. The explant methodology worked to isolate the DPSCs without using any enzyme that could potentially alter their properties^47^. Kerkis et al. used the explant culture method to obtain DPSCs from deciduous teeth. The DPSCs were propagated in α-MEM with 15% FBS. The cells were fibroblast-like morphology and were able to differentiate into osteoblasts and adipocytes which exhibited their multipotency^61^. In the extraction of DPSCs from human third molars, the current report used mechanical mincing and enzymatic digestion with collagenase and dispase. Cells were cultured using DMEM, 10% FBS and antibiotics. This combined approach was found to be superior for extracting a greater number of viable DPSCs^62^. Zhang et al. used both enzymatic digestion and mechanical dissociation for isolating DPSCs. The cells were subsequently isolated using magnetic-activated cell sorting (MACS) based on expression of stem cell markers such as STRO-1. This method allowed for the isolation of a more consistent population of stem cells that have an increased potential for regeneration^62^. Zhang et al.^18,62^ studied the cryopreservation of DPSCs and demonstrated that DPSCs that were cryopreserved were still viable and able to divide and differentiate into multiple cell types. This study illustrated the importance of having cryopreservation of DPSCs to use in a cell banking system and to study different applications^54,63,64^. This study utilized DPSCs obtained from human third molars and demonstrated that DPSCs could differentiate towards cell lineages such as odontoblasts, osteoblasts and adipocytes. In addition to cell fate and differentiation capacity, Zhang et al.^61^ also used an experimental culture medium with growth hormones to increase differentiation capacity of DPSCs. Huang et al. cultured DPSCs in a not fully defined condition thought to induce differentiation into neuronal cell types. They also demonstrated that DPSCs could differentiate towards neuronal cell lineages by expressing neural markers, which may provide potential applications in the areas of brain regeneration and dental tissue engineering^65^. This research studied the gene expression profile of DPSCs sourced from human teeth. The cells were grown in DMEM + 10% FBS and found to express a unique gene profile related to stem cell maintenance, differentiation and secretion of an extracellular matrix^48^. Arthur et al. conducted a comparative study of DPSCs and bone marrow–derived mesenchymal stem cells. DPSCs were grown in conventional stem cell medium and their gene expression profile was studied to identify uniquely dental pulp stem cell markers that distinguished them from other types of MSCs^50^. This study explored the use of 3D scaffolds to grow DPSCs for the purpose of dental tissue engineering. The DPSCs were seeded on biodegradable scaffolds and cultured in a bioreactor system in which they differentiated into odontoblast-like cells and formed dentin-like structures^66^. Piva et al.^66^ created a co-culture method in which DPSCs grew alongside endothelial cells to increase vascularization in engineered tissue constructs. The study demonstrated that DPSCs can develop into odontoblasts and endothelial-like cells indicating their role in the development of the dental pulp and its supporting vasculature^67-69^. The number of methods to generate and propagate DPSCs is extensive. This underlies their versatility and adaptability to an array of experimental conditions^70^. Each method has its merits and limitations, and usually the method chosen corresponds to the research objectives^71^. As the field of DPSC research progresses, these methods are likely to continue to improve, thus increasing the ways we can apply the regenerative abilities of DPSCs for therapeutics^72^.

**Figure 1.**
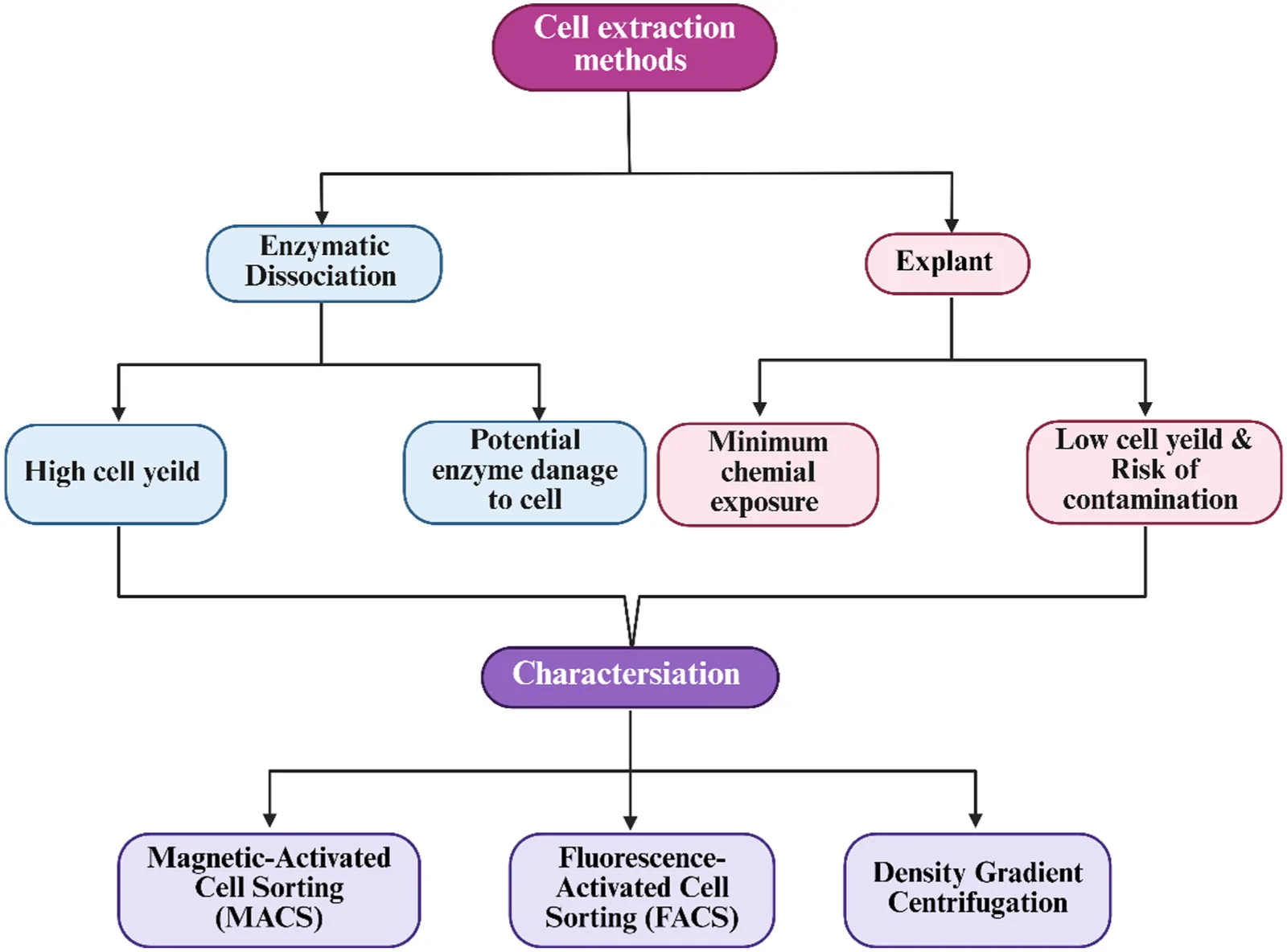
Cell extraction methods and characterization (Figure created by the authors using BioRender.com).

### Therapeutic applications and limitations

The application of DPSCs can potentially employ direct cells indistinctly in targeting therapies to regenerate dental pulp, where they could differentiate into odontoblast-like cells to produce dentin-like materials^66,68^. This may be more effective than for localized injuries as well^73^. The use of the DPSC secretome has additional thoughts beyond dental applications. This has helped in the treatment for neurodegenerative diseases, cartilage defects and bone regeneration due to its anti-inflammatory and neuroprotective effects^22,23,43,74^. To help support this conclusion, studies have explicitly shown that the DPSC-derived secretome results in neurite outgrowth in neuronal cultures and, thus, may have benefits in neurological therapies^75,76^ (Fig. 2).

**Figure 2.**
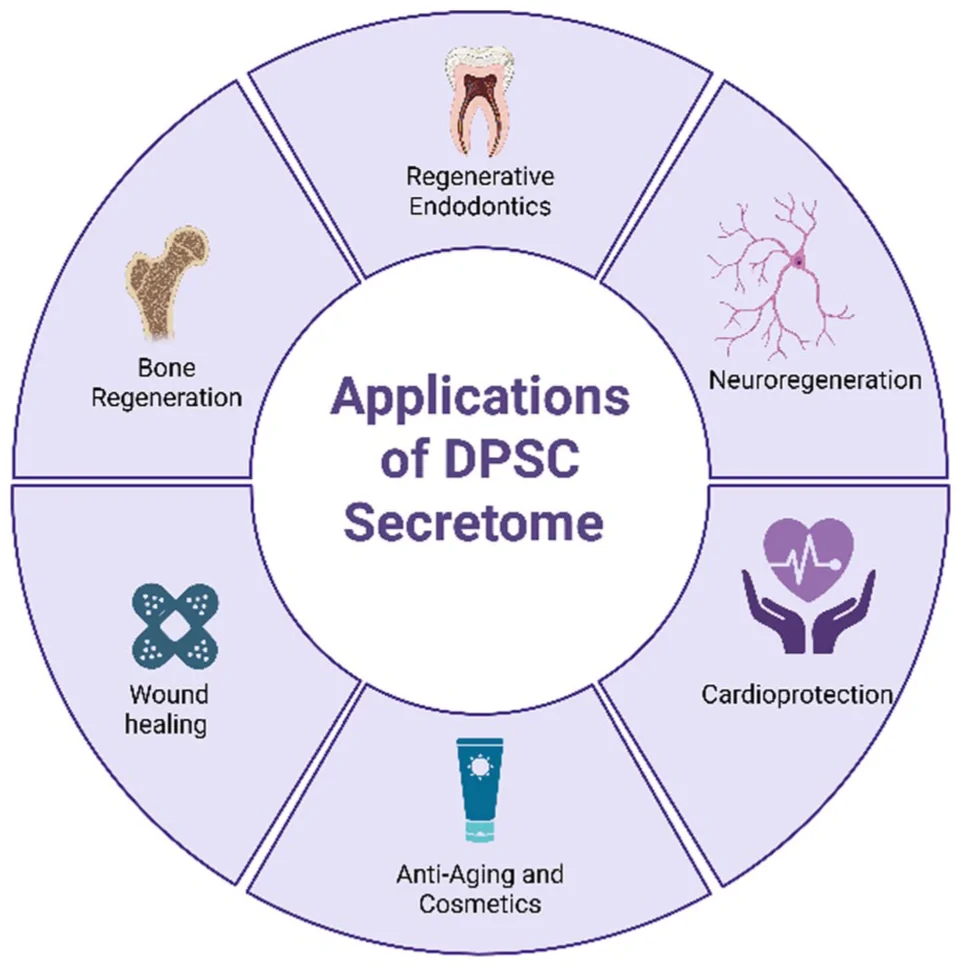
Application of DPSC secretome (Figure created by the authors using BioRender.com).

### Advantages and limitations of using DPSCs

Directly using DPSCs allows for immediate cellular activity at the injury site^69^. The ability of DPSCs to differentiate into multiple cell types can be harnessed for complex tissue structures^20^. Limitation being that there is a risk of tumorigenesis as introducing live cells carries risks associated with uncontrolled growth or differentiation^73,77^. Logistical challenges such as cell-based therapies require more rigorous handling protocols compared to using a concentrated secretome^24,78^.

### Role of scaffolds in pulp regeneration

Scaffolds have a diverse multifaceted function during dental pulp regeneration^79,80^. They serve as a three-dimensional structure that supports cell adhesion and growth^81^. Scaffolds can affect cellular behaviour through biochemical signals that induce stem cell differentiation into certain lineages important for pulp tissue development^71,82^. Successful pulp regeneration includes the development of a vascular network to provide nutrients and oxygen to the newly formed tissues and initiate vascularization^58,83^. Depending on the design and incorporation of angiogenic factors, scaffolds can also promote angiogenesis^84,85^. Scaffolds can be seeded with dental pulp stem cells (DPSCs) or other stem cells so that the repair tissue is established in an environment conducive for repair^22,59^. Innovative scaffold technologies are also now being investigated to enhance functionality^71^. Nanofibrous scaffolds are being developed from electrospinning techniques that produce nanofibrous scaffolds meant to enhance the functionality of scaffolds and best simulate the architecture of natural extracellular matrix (ECM) which may enhance cell adhesion and proliferation^61,86^. Hydrogel-based scaffolds would facilitate injectable hydrogels that are also becoming popularized due to their ease of application in clinical environments, their unique ability to encapsulate cells and bioactive factors, and minimize the invasiveness of therapeutic procedures while promoting regeneration^85,87–89^. Antibiotic-impregnated scaffolds are being studied for use in dentin pulp to minimize the risk of infection associated with regenerative procedures and significantly improve the outcomes of healing and regeneration^90^. The advancement of successful scaffolds for regeneration of dental pulp has become a promising field of research that could benefit regenerative dentistry^91^. In utilizing various types of scaffolds (e.g. natural, synthetic, bioceramic and composite), researchers are working to establish the best possible environment for pulp healing and regeneration, along with understanding the materials’ specific properties and roles as scaffolds^92^. Future research should continue to explore advanced materials and fabrication strategies to improve scaffold performance while keeping safety and efficacy in mind for clinical use. Another exciting area of interest is the inclusion of bioactive molecules in scaffolding systems as part of strategy for improving dental pulp regeneration. In the case of using DPSCs on biological scaffolds – scaffolds composed of natural matrices such as collagen or decellularized tissue – tissue integration may improve since DPSCs have been found to secrete extracellular matrix components that permit cellular adhesion and migration. Scaffolds that include DPSC secretome may further help support functional integration of scaffolds by providing certain growth factors to the tissue that promote angiogenesis and recruitment of cells. Studies comparing scaffolds combined with DPSC secretome showed improved regeneration and vascularization than scaffolds alone, or scaffolds populated just with DPSCs^24,80,93^ (Table 2).

**Table 2.** Comparison studies between DPSC-based therapies and cell-free therapies.

Category	Therapy / strategy	Current status	References
DPSC-based therapies:			
Autologous DPSC transplantation + G-CSF + Scaffold	Transplantation of patient’s own DPSCs into disinfected root canal with G-CSF-loaded collagen scaffold	Clinical trials (Japan) successful regeneration of vascularized, innervated pulp tissue	Nakashima et al.^94^
Allogeneic DPSC transplantation	Use of banked DPSCs from other individuals with scaffold support	Preclinical & early-phase clinical studies	Iohara et al.^95^
DPSC-seeded scaffolds (e.g. collagen, fibrin, hyaluronic acid)	3D scaffolds seeded with DPSCs for pulp regeneration in animal models and *ex vivo* roots	Preclinical (animal studies)	Hsiao et al.^96^
Cell-free therapies:			
Cell-free therapy (DPSC/SHED secretome or exosomes)	Use of conditioned media or exosomes from DPSCs to promote regeneration without live cells	Preclinical (animal and *in vitro* models)	Ganesh et al.^97^
DPSC/SHED secretome	Use of conditioned media containing soluble factors (growth factors, cytokines, chemokines) from DPSCs or SHED to promote pulp regeneration without using live cells.	Preclinical (animal and *in vitro* models).	Sarra et al.^98^
DPSC/SHED-derived exosomes	Use of nano-vesicles enriched with proteins, lipids and miRNAs released by DPSCs/SHED to stimulate angiogenesis, cell proliferation and dentin–pulp repair.	Preclinical (rodent and *in vitro* regeneration assays).	Li and Ge^99^ and Chansaenroj et al.^100^

## Dental pulp stem cells secretome

### Cell-free DPSC secretome

In recent years, there has been a promising shift in regenerative endodontics which involves the adoption of cell-free strategies, particularly those utilizing the secretome derived from DPSCs. Amid various MSCs subtypes, dental pulp stem cells have been found to have a wide range of sources and low immunogenicity and exhibits significant regenerative advantages over bone marrow mesenchymal stem cells, including enhanced proliferative kinetics, marked osteo-inductive capacity and dual angiogenic and immunomodulatory paracrine secretion profiles^20,53,101,102^. The DPSC- derived secretome often collected as conditioned medium (CM) is composed of a diverse array of bioactive components, such as growth factors, cytokines, chemokines, extracellular vesicles (EVs) and exosomes^73,103^. These collectively mediate tissue repair through paracrine mechanisms, making them highly valuable in regenerative medicine applications^104^. Conditioned medium is typically harvested after culturing DPSCs in serum-free or reduced-serum medium for 24–72 h, depending on the experimental protocol^105,106^. Several optimization studies have highlighted the influence of culture duration, oxygen tension and serum starvation on the bioactivity of the DPSC secretome^107,108^. Nevertheless, studies directly investigating the therapeutic potential of DPSC-derived secretome in dentin–pulp tissue regeneration and modulation of inflammatory responses are still relatively scarce^101,103^. Some of the preliminary studies that have been explored where the secretome was collected from hDPSCs exhibited anti-inflammatory effects and promoted odontoblastic differentiation, and it was also identified that these effects mediated through MAPK and PI3K/Akt signalling pathways^52^. Furthermore, DPSC-CM has shown the ability to modulate macrophage polarization, reduce oxidative stress and enhance angiogenesis *in vitro* and *in vivo*^109,110^. There are numerous studies where they have explored various applications of DPSC secretome in various fields such as neuroregeneration, wound healing and cardiovascular repair; its integration into regenerative endodontics remains underexplored and holds significant promise. Table 2 provides a comparative overview of DPSC-based cellular therapies and cell-free approaches in context of endodontic regenerative medicine.

### DPSC secretome: definition and composition

The secretome plays a key role in improving the therapeutic effects of dental pulp stem cells (DPSC)^75,111^. Conditioned medium (CM), also called the secretome, consists of diverse bioactive molecules, including three well-recognized growth factors (BMP-2, VEGF and IGF-1), which regulates several cellular functions, including survival, proliferation, migration and differentiation. The secretome modulates the local microenvironments by supporting angiogenesis and reducing inflammation, which are important for tissue regeneration^41,102,112^. There is growing evidence that DPSC-derived conditioned medium (CM) significantly increases the proliferation and differentiation associated with many cells^113,114^. The capacity of DPSCs to stimulate a regenerative response makes them valuable in regenerative treatment strategies, especially for dental and craniofacial tissues^70,115^. The secretome consists of bioactive molecules that are secreted from DPSCs, growth factors, cytokines and extracellular vesicles (EVs)^72,73,116^. Those components are essential in mediating the paracrine effects of DPSCs to promote or facilitate the process of tissue regeneration^117^. The secretome can also regulate biological responses such as angiogenesis, modulation to inflammation and cell survival^76,118^. Formidable secretome can be obtained from cultured DPSCs using CM and EVs, which makes the secretome an advantageous therapeutic option independent of cell-based transplantation^119^. Through a paracrine signalling mechanism, the secretome can signal to neighbouring cell types and the local microenvironment through bioactive factors released by DPSCs^120^. The secretome has the potential to enhance healing without requiring the direct presence of DPSCs at the wound site^93,121^. This occurs in circumstances which make cell transplantation risky due to the potential for immune rejection of the transplanted cells or the potential increased risk of tumour formation^122,123^.

### Isolation and complexity of DPSC secretome

The secretome of dental pulp stem cells (DPSCs) refers to a complex and diverse mixture of proteins, growth factors, cytokines and extracellular vesicles that are secreted by DPSCs. The isolation technique of DPSC secretome is when the DPSC cells are cultured in serum free or low serum for 24–72 h once the cells reach the confluency of 70%–80%. The conditioned medium is collected and clarified by sequential centrifugation (low-speed spins to remove cells/debris), filtered and optionally concentrated (ultrafiltration) prior to its usage (centrifugation ranges: 300 × g–2000 × g). The isolation of EV/Exosome isolation is commonly performed by differential ultracentrifugation, size-exclusion chromatography or polyethylene glycol (PEG) precipitation^21,73^. Culture preconditioning like hypoxia, inflammatory stimulation or growth factor priming modifies secretome composition and can be exploited to enrich desired bioactive factors.^20^ Recently, the DPSC secretome has gained popularity in regenerative medicine, particularly in dental applications, and more widely, for wound healing and neuroprotection. Vascular endothelial growth factor (VEGF) stimulates angiogenesis and vascularization, key to generating new blood vessels in tissues being regenerated^69^ Transforming growth factor-beta (TGF-β) stimulates cell differentiation, proliferation and immune response and is essential to differentiate DPSCs into odontoblast-like cells^68^. Bone morphogenetic proteins (BMPs), especially BMP-2 and BMP-4 signalling, is key to osteogenic differentiation of DPSCs and dentin and bone formation^124^. Fibroblast growth factors (FGFs) such as FGF-2 stimulate stem cell proliferation and differentiation, DPSCs inclusive. They also serve to induce angiogenesis and are important for healing^24^. Interleukin-6 (IL-6) is a pro-inflammatory cytokine with some anti-inflammatory effects, depending on the context. IL-6 plays a role in immune regulation and can promote tissue survival and regeneration^125^. Interleukin-10 (IL-10) is an anti-inflammatory cytokine that provides regulation of the immune response to limit excessive tissue damage during inflammation^125,126^. Tumour necrosis factor-alpha (TNF-α), while associated with inflammation, can play a role in promoting tissue regeneration, in addition to its modulation of various cell type behaviour^23^. Exosomes are small vesicles (30–150 nm) that can transfer a variety of bioactive molecules, including proteins, lipids and RNAs that modulate target cell behaviour. Exosomes from DPSCs promote angiogenesis, osteogenesis and neuroprotection^21,75,125,126^. Macrovesicles are larger vesicles (100–1000 nm) that can also transfer bioactive molecules between cells that contribute to tissue repair and regeneration^126,127^. Matrix metalloproteinases (MMPs) MMP-2 and MMP-9 are enzymes capable of degrading the extracellular matrix (ECM) and involved in tissue remodelling in regeneration. They also contribute to the migration and differentiation of stem cells^75^. HSP70 and HSP90 (heat shock proteins) assist in protecting cells from stress and participate in the correct folding and functioning of other proteins. They have also been implicated in promoting cell survival and tissue regeneration^124^. Collagen Type I and III complete the extracellular matrix and play an important role in the structural integrity and function of the regenerating tissues^24^. The DPSC secretome has gained considerable traction in a variety of fields, examples being dentin regeneration – the DPSC secretome has been shown to promote the differentiation of DPSCs into odontoblast-like cells, resulting in the formation of dentin-like structures. A key component for this process is the presence of growth factors such as TGF-β and BMPs in the secretome^40^. The secretome can promote the survival and proliferation of DPSCs and other cells within the dental pulp to promote the regeneration of pulp tissue^40^. The DPSC secretome has been studied for its ability to regenerate periodontal tissues such as the periodontal ligament and alveolar bone^50^. The growth factors and cytokines present in the DPSC secretome (e.g. VEGF and IL-6) encourage angiogenesis and tissue repair; therefore, it may be a useful strategy to facilitate wound healing in the skin^75^. Several recent studies have evaluated the ability of the DPSC secretome to accelerate healing following a corneal wound, through enhancing epithelial cell migration and decreasing inflammation^57,104^. DPSC secretome contains growth factors and cytokines that promote the survival, proliferation and differentiation of neural cells, which may potentially be utilized for neurodegenerative diseases or to repair nervous tissue following injury^40^. The DPSC secretome has become a focus of inquiry due to its neuroprotective properties, which can facilitate cellular recovery and reduce damage following stroke or traumatic brain injury^123^. Potentially, there is ability for new blood vessel formation by the VEGF and other pro-angiogenic factors present in the DPSC secretome, which would be highly beneficial in the repair of ischemic injured tissues^77^. There is an emerging medical interest in using the DPSC secretome to promote regenerative abilities in injured cardiac tissue, following a myocardial infarction, using its vascularizing effects and ability to contribute to reduced fibrosis^126,128^. This study characterized the proteome of the DPSC secretome and identified numerous proteins involved in tissue regeneration and immune modulation. The authors highlighted the potential of the DPSC secretome in regenerative medicine, particularly for dentin and pulp regeneration^126,127^. Martens et al. investigated the neuroprotective effects of the DPSC secretome in a rat model of stroke. It was also found that the secretome can considerably reduce infract area and enhance functional recovery, thus indicating the potential of utilizing the secretome therapeutically in neurological disorders. This investigation concentrated on the angiogenic action of the DPSC secretome. the authors found that exosomes derived from DPSCs elicited new blood vessel development both *in vitro* and *in vivo* which further supports its application in tissue engineering and regenerative medicine^129^. Hilkens and co-workers investigated the potential regenerative properties of the DPSC secretome on periodontium regeneration and found that the secretome can significantly enhance the proliferation and migratory properties of periodontal ligament cells to stimulate regenerated periodontal tissues. This research investigated the use of DPSC secretome for corneal wound healing^68^. Their findings showed that secretome can not only speed epithelial wound closure but also reduce inflammation, indicating its potential for treating ocular surface injuries. Previous studies have shown that subjecting DPSCs to hypoxic conditions (low oxygen levels) before harvesting the secretome can enhance the secretion of pro-angiogenic and anti-inflammatory factors, improving its therapeutic efficacy. Adding specific growth factors to the culture medium of DPSCs can enhance the production of desired components in the secretome, such as increasing the levels of VEGF for better angiogenesis. Applying mechanical forces to DPSC cultures can modulate the secretion profile, enhancing the production of proteins involved in tissue remodelling and regeneration^118^. The DPSC secretome represents a powerful and versatile tool in regenerative medicine, with applications ranging from dental tissue engineering to neuroprotection and wound healing^130^. The ongoing research into optimizing the secretome through preconditioning and other methods holds great promise for developing more effective and targeted therapies^118^. The studies referenced here provide a strong foundation for further exploration and application of the DPSC secretome in various clinical contexts. Characterization of the secretome composition is analyzed using various protein assays (BCA, ELISA, LC-MS/MS), western Blot markers (CD63, CD81, TSG101), functional assays for angiogenesis (tube formation), mineralization (ALP, DSPP expression) and anti-inflammatory effects (IL-10/IL-6 ratio). Culture conditions such as oxygen tension, glucose levels and mechanical stress significantly affect secretome yield and bioactivity^73,101,126,131^. The conditioned medium obtained from DPSCs also enhance odontogenic markers (DSPP, DMP-1, RUNX2) in primary dental pulp cells and stimulates mineralized matrix deposition *in vitro*. Conditioned medium from DPSCs enhance proliferation and odontoblastic differentiation of human pulp cells, up-regulating DSPP and RUNX2. It also induces endothelial tube formation through VEGF-dependent pathways and supresses pro-inflammatory cytokines in macrophage cultures. There has recently been an emphasis on improving the extraction and characterization of DPSC secretome, with the intent to maximize their regenerative potential^113^. High-performance liquid chromatography (HPLC) and mass spectrometry are being utilized to characterize the aspects of the secretome^117,126,132^. Understanding the various components of the secretome will pave the way for personalized medicines that utilize specific components to induce tissue regeneration^19^. Additionally, there are scientists who determine how various culture conditions affect the composition of the DPSC secretome. The secretion profile has been shown to be influenced by environmental factors such as oxygen tension and nutrient availability, which also influence the efficacy of treatment^115,125,133^. The study of DPSCs and their secretome represents an emerging area of research in regenerative medicine^82,130,131^. Researchers have ambitions to improve approaches for dental pulp regeneration, and possibly other forms of tissue repair, by utilizing both DPSCs and the bioactive secretome that DPSCs produce in a 3D scaffold. Research continues to reveal the complexities of DPSC biology and their secretome, and it is anticipated that new research will enable various forms of therapeutic use that would enhance important outcomes related to regenerative therapies^133^.

### Secretome-functionalized scaffolds: delivery strategies for the root canal

Scaffolds provide mechanical support and controlled delivery for secretome components. Hydrogels such as GelMA, collagen, chitosan/alginate, injectable matrices and composite scaffolds have been studied as carriers for CM and EVs^103,122,134,135^. Key design goals include sustained release of bioactive factors, preservation of EV integrity, porosity for cell and vessel ingrowth, and ease of clinical delivery into the root canal space^135^. Studies demonstrate that secretome-loaded hydrogels enhance angiogenesis and odontogenesis versus unloaded scaffolds, and structuring scaffolds with microchannels improves nutrient diffusion and vascular penetration^127,131,136,137^. Translation-ready formulations will repair scalable manufacturing and validated release profiles^131^.

### Advantages and limitations of using DPSC secretome

Since it does not contain live cells, the utilization of the secretome alleviates concern for immune response^39^. The secretome can be harvested and subsequently stored (frozen) to provide flexibility in the application of the therapy^39^. The bioactive factors present within the secretome would be capable of targeting multiple pathways associated with healing across multiple types of tissues. The complexity of the secretome composition may vary and depend on conditioning of the culture conditions which may affect the efficacy of the secretome^23^. While they may be affective promoting healing via paracrine signalling, the lack of live cells limits some regenerative processes that require live cell to cell interactions^20^. While there are benefits from utilizing DPSCs and their secretome within regenerative medicine compared to one another, there are some unique advantages from each perspective. Direct use of DPSCs can be beneficial in specifically treating localized regeneration as you would be infusing live cells, while using their secretome provide a flexible approach to promote healing by only using bioactive signalling while perhaps avoiding some potential risks associated with live cells. Future work should explore the scaffold design that incorporates a combination of live DPSCs and their secretome to promote the best regenerative improvements minimizing the associated risks^123^. Understanding the abilities to combine these two strategies can provide clinicians with valuable information to deter treatments for both dental pulp regeneration and to encourage tissue repair in other medical fields.

## 3D bioprinted products in endodontic procedures

Endodontics is a specialized branch of dentistry that deals with the treatment of dental pulp and periapical tissues. With the advent of 3D bioprinting technology, strategies have been transformed to allow some bioprinted products to address some complex challenges in pulp regeneration, root canal therapy and tissue engineering^13,87^. This transformation is moving the spectre of health care away from conventional practices to patient-centric solutions that combine precision and biocompatibility in contemporary dental care^88^. Bioprinting is a specific category of 3D printing that utilizes bioinks containing living cells and biomaterials^12^. This capability allows for complex structures that mimic the natural organization of dental tissues^87^. The promise of bioprinted products in endodontics is their capacity to reproduce the native environment of the dental pulp to allow regeneration, repair and healing^138^. As the role of 3D Bioprinting in Endodontics title demonstrates, 3D bioprinting has been a game changer in dental care. It gives rise to solutions in endodontics for traditionally unresolved complexities such as pulp necrosis, periapical lesions and the repair of damaged root canals. Unlike traditional treatment protocols that use inert materials, bioprinted products strive to restore the physiologic function of dental tissue^138^. The function of 3D bioprinting is an important aspect of endodontics, particularly in the development of scaffolds used for dental pulp regeneration that enable cell attachment, proliferation and differentiation and allow new tissue to grow^139^. The exact control over the layer-by-layer deposition of bioinks allows for a scaffold architecture that approximates native pulp tissue architecture to improve function. Bioinks are the most essential element of 3D bioprinting. In endodontic applications, bioinks must meet a lot of criteria, including biocompatibility, mechanical strength and the ability to support cellular activity^140^. Bioinks are commonly hydrogels – different types that can be used to have excellent biocompatibility and tuneable properties are gelatin, alginate and collagen-based hydrogels^11^. In terms of synthetic polymers, materials like polyethylene glycol (PEG) and polylactic-*co*-glycolic acid (PLGA) have some structural integrity and are biocompatible^123^. Cell-laden Bioinks is the application of dental pulp stem cells (DPSCs) or mesenchymal stem cells (MSCs) in bioink formulations that can enhance their regenerative properties^140^. The further development of bioink formulations has generated multi-material scaffolds that mimic the heterogeneous nature of dental tissues^134^. The bioinks can be configured to also release bioactive molecules like growth factors as additional stimuli for tissue regeneration^139^. An area of particular interest is the regeneration of pulp tissue, a key aspect of endodontics. Current management of pulp necrosis usually involves the removal of infected tissue and filling of the canal space with an inert material without allowing for the biological function of the pulp to be restored^68^. The use of bioprinted scaffolds is a potential solution to this problem^139^. These scaffolds are designed to reproduce the native extracellular matrix (ECM) of dental pulp and provide a growth environment for cells^139,140^. In addition to the structural support and cell attachment surface, the scaffolds can be modified to include microchannels, which encourage a vascular pathway, which is essential for successful pulp regeneration. There are evidence-based studies that utilize scaffolds with DPSCs and upon implantation into root canals develop vascularized pulp-like tissue and overall better treatment outcomes^67^. Furthermore, the addition of antimicrobial agents to the scaffold matrix can help to eliminate the potential for reinfection. The conditioned medium from DPSCs enhances proliferation and odontoblastic differentiation of human pulp cells, up-regulating DSPP and RUNX2. It also induces endothelial tube formation through VEGF-dependent pathways and supresses pro-inflammatory cytokines in macrophages culture^131,141^. In rat and canine models of pulp exposure, DPSC-secretome injection or secretome-loaded scaffolds produced continuous dentin-like tissue, increased vascular density and reduced inflammatory infiltration. Neurotrophic factors within the secretome facilitated nerve regeneration within the pulp chamber. In early-phase clinical trials are evaluating the safety and efficacy of DPSC-secretome or exosome-based scaffolds in human teeth with necrotic pulps. Preliminary outcomes indicate biocompatibility and formation of vascularized pulp-like tissue without adverse effects. Although many preclinical studies show the effectiveness of DPSC-loaded scaffolds for pulp regeneration, clinical translation remains limited. Most registered clinical trials evaluate DPSCs without scaffold-based delivery or are focused on periodontal regeneration rather than pulp tissue engineering.^142^ (Table 3).

**Table 3.** Existing clinical trials and Pre-clinical studies of DPSC scaffolds.

Pre-clinical & Clinical trial	Objective	Scaffold material	Phase	Status	References
Pre-Clinical studies	Evaluate the safety and efficacy of DPSC-based scaffolds for pulp regeneration	Collagen-based scaffold	-	Ongoing	Han et al.^143^
Pre-Cilnical studies	Assess pulp–dentin complex regeneration using DPSC-loaded hydrogel scaffolds	Hydrogel-based scaffold	-	Recruiting	Hsueh et al.^137,144^
Clinical Trial ID: NCT03386877	Periodontal Regenration Using Dental Pulp Stem cells (DPSCs)	Collagen based scaffold	NA	Completed	ClinicalTrials.gov^145^
Clinical Trial ID: NCT06006052	Regenrative Endodontic in Immmature Permanent teeth	Deminarlized Dentin Matrix	NA	Unspecified	ClinicalTrials.gov^146^
Clinical Trial ID: NCT05924373	Human Mesenchymal Dental pulp stem cells for preiodontal regenration	Stem Cell Injection	Phase II	Recruiting	ClinicalTrials.gov^147^

## Future prospectives of 3D bioprinting techniques for endodontics

Various 3D bioprinting techniques are utilized to fabricate endodontic products. The ideal one being extrusion-based bioprinting as it prints scaffolds with high cell density. Stereolithography (SLA), using photopolymerizable materials, produces highly detailed structures suitable for intricate root canal geometries^135^. Inkjet Bioprinting enables the deposition of small droplets of bioink, allowing for the creation of complex tissue patterns. Emerging technologies, such as four-dimensional (4D) bioprinting, hold promise for developing dynamic scaffolds that can adapt to the changing environment within the root canal^135^. The applications of 3D bioprinted products in endodontics are diverse. Stem cell bioprinted scaffolds restore biological function of the pulp as they promote healing and minimize the risk of secondary infections^33^. Custom-made scaffolds promote regeneration of the periodontal ligament, the specific structure that anchors teeth to the alveolar bone^36^. Bioprinted constructs induce the formation of tissue at the root apex and allow closure of immature teeth. Bioprinted scaffolds may be useful in repairing tissues affected by periapical infections^148^. While there are many potential uses, the transition to clinical practice is challenged by numerous hurdles, such as regulation to ensure that bioprinted products meet the standard required to be eligible for clinical use. There are also limitations in terms of technology, as well as the fact that the materials needed are expensive and don’t allow for smooth accessibility for everyone. Most importantly, though, the viability and functionality of bioprinted tissues in the long term is still a significant challenge. Research is presently underway to overcome these barriers in order to improve the scalability and affordability of bioprinted products. Indeed, the future of bioprinting in endodontics is promising, and this is only possible by increasing improvements in technology as well as improving the materials to move innovation forward. Thus, the creation of an entire tooth – with pulp, dentin and enamel layers – could be possible. The bioprinted products created could be customized specifically to individual patient needs. Additionally, artificial intelligence could be used to create optimized scaffold designs for the bioprinted constructs while also utilizing technical intelligence data to predict the results of treatment. The evolution of bioprinting will likely offer bioprinted products as a component of endodontic care, which can contribute to better outcomes and overall satisfaction for patients. The worldwide market for 3D-printed dental implants is growing due to developments in additive manufacturing, demand for custom dental products and growing prevalence of dental disorders.

## Market size and regulatory parameters

### Market size of 3D printed dental products

The global dental 3D printing market was valued at about $2.9 billion in 2023 and is projected to reach $8.1 billion by 2029, at a compound annual growth rate (CAGR) of approximately 19%^149,150^. North America was the largest region of the 3D dental printing market in 2023, capturing about 38.8% of total revenues due to its high health care expenditure, low rates of uninsured people and overall advancements in technology, while the Asia-Pacific region is expected to grow quickly with a projected CAGR of 27.1%, primarily due to rising dental tourism, improvements in healthcare infrastructure and higher disposable income. Crowns, bridges, dentures and implants are common dental products that are 3D printed^150^. These products can fulfil multiple purposes, including restorative procedures, orthodontics and prosthodontics. The more widely used technologies in the industry are, stereolithography (SLA), selective laser sintering (SLS) and fused deposition modelling (FDM). SLA is known for its superior precision, while the other processes offer more affordable solutions. When technology can provide dental restorations with high levels of customization that can meet individual patients’ needs, one major advantage is improved clinical results. Furthermore, the increase of aesthetic dental options and products has contributed to the adaptation of new innovations in dentistry^151^. The increase in edentulousness, or total tooth loss among older adults, has also contributed to an increase in the need for dental prosthetics or dental implants. The high costs of equipment, as well as a lack of trained professionals to carry out the procedures, are barriers to broader acceptance. Regulatory hurdles to manufacturers due to regulations are difficult for manufacturing firms to comply with. Emerging markets, such as China, India and Brazil, provide enormous growth opportunities as access to healthcare expands and with benefits from dental tourism. Key Players and Innovations Major players include Straumann (Switzerland), Dentsply Sirona (USA), Zimmer Biomet (USA) and 3M (USA). Recent innovations such as Stratasys’s True Dent, which is a full-colour 3D-printed denture, and Align Technology, which has received FDA approval for its Invisalign system, not only demonstrate further advancement of efficiency in the industry but further prove that the market for dental implants and prosthetics is primed for disruption. Therefore, the 3D dental implant market is expected to expand, due to continued technological advancement and increased focus on personalized healthcare. The global market for 3D bioprinting in regenerative medicine, including bioprinted scaffolds with integrated advanced biological components, such as Dental Pulp Stem Cells (DPSCs) and the DPSCs secretome, is experiencing significant growth. In 2023, the 3D bioprinting industry is estimated to be around USD 1.8 billion, with significant contributions from dental and craniofacial applications. The increasing demand for personalized and regenerative dental therapies due to advantages in tissue engineering and stem cell research has positioned DPSCs as a key player in the market development. DPSCs are attractive due to their easy separation, minimally invasive procedure and regenerative properties. The secretome produced from DPSCs consists of bioactive molecules, including cytokines, growth factors and extracellular vesicles, that aid in regenerative medicine. The secretome enhances the structural and functional properties of scaffolds manufactured using 3D bioprinting. The combination of these technologies can lead to the formation of biomimetic solutions that address complex dental tissue defects and facilitate market uptake. The global exosome therapeutics market which covers exosome or secretome products from mesenchymal and stem cell sources is projected to expand rapidly, with showing some estimated growth from hundered of millions of USD in the mid-2020s to well over USD 800 million by 2030^149^. Usually, DPSC secretome integrated scaffolds would stand to benefit from these market trends. Given the rising investment in regenerative medicine and exosome technologies, DPSC secretome therapies have a realistic potential to evolve from preclinical research into scalable, off-the-shelf dental products within the next 5–10 years^150^. Multiple drivers support the market growth of 3D bioprinted scaffolds using DPSCs and their secretome. An increasing prevalence of diagnosed dental diseases, such as dental caries, pulp necrosis and periodontal diseases, is common leading to an increased demand for regenerative therapies to restore pulp–dentin function^152^. The development of 3D bioprinting capabilities will allow an expansion of potential integration of stem cells and secretome into scaffold design, with the development of multi-material bioprinters, as well as scaffolds engineered with precision. Investment in research and development surrounding stem cell-based therapies is funded by the government and private investors, and when fully funded, the commercialization of DPSC-integrated scaffolds can be facilitated. The use of less invasive, non-surgical regenerative procedures is increasing with potential for less recovery time or better patient outcomes after the use of non-surgical procedures, further driving demand. Progressive regulatory frameworks and approvals for therapies based on cells and those that use scaffolds for support are opening up pathways for innovative products to enter the marketplace^149^.

### Market growth projections

The 3D bioprinting sector is forecasted to expand at a compound annual growth rate (CAGR) of 15%–20% between the years 2023 and 2030, with an estimated market value between USD 6 and 8 billion by the conclusion of the forecasted period^151,153^. The 3D bioprinting domain that concentrates on DPSC-based scaffolds and secretome is anticipated to expand at a quicker pace due to greater clinical acceptance; the more preclinical and clinical trials demonstrate safety and efficacy, and it is likely that they will be accepted into routine dental treatment. Together with growth factors, nanomaterials and controlled-releasing systems, DPSCs and their secretome will yield greater therapeutic outcomes, making them more appealing to clinicians and patients. The emerging economies of Asia-Pacific, Latin America and the Middle East are investing heavily in health care infrastructure and regenerative medicine markets; these are open opportunities for market penetration^153^. Various companies and research institutions are currently innovating in 3D bioprinting and stem cell–based regenerative therapies. Some these include key players such as Organovo Holdings, CELLINK and Aspect Biosystems, along with new dental regeneration start-ups^153–155^. Partnerships between bioprinting and start-up companies, educational institutions and health care providers will help to innovate delivery and commercialization of DPSCs. Although there are challenges ahead, the market has a lot of potential for growth because of the same challenges. High production costs, complex regulations and the need for strong clinical data will be some challenges. However, innovations can address these obstacles, including advancements in cost-effective production techniques, improvement to scaffold design and improved protocols for secretome isolation and delivery systems. The market for 3D bioprinted scaffolds containing DPSCs and their secretome is positioned for growth and transformation from the combination of technological advancements, the growing perception of regenerative therapies in medicine and the increase in patient demand for individualized dental treatment. As research and development begin to address existing challenges, this niche market will play a significant role in regenerative dentistry and beyond.

### Regulatory concerns

The clinical translation of DPSCs and their secretome faces significant regulatory challenges that must be addressed to ensure safety, efficacy and compliance. The classification of these biologics is a major source of concern; depending on the jurisdiction, DPSCs are usually regulated as human cells, tissues, and cellular and tissue-based products or advanced therapy medicinal products^140,156–158^. The secretome, which includes cytokines, growth factors and extracellular vesicles, is frequently handled as a biologic or combination product, which raises concerns regarding potency assays, batch consistency and standardization^140,157,158^. Exosomes therapeutics are attracting significant commercial interest across medical fields. Regulatory agencies emphasize product characterization and reproducibility for EV-based products, so early engagement with regulatory authorities is recommended for translational programs. Robust characterization, including thorough composition analysis, stability data and proof of mechanism of action, is required by regulatory bodies like the FDA and EMA. Because of their intricate structure and the requirement for repeatable isolation and purification techniques, extracellular vesicle-vesicle-based products in particular continue to be regulated^159^. Moreover, both cell-based and cell-free (secretome) have to conform to Good Manufacturing Practice (GMP) in order to ensure patient safety, particularly when considering risks of immune rejection, contamination and potential tumorigenicity in long-term applications^140,160^. Ethical considerations also arise from the use of donor-derived stem cells, particularly in cross-border applications, necessitating harmonized international guidelines for sourcing, traceability and informed consent^161^. These regulatory and ethical hurdles must be navigated carefully to facilitate successful commercialization and clinical adoption of DPSC- and secretome-based therapies for dental pulp regeneration. Beyond regulatory compliance, the successful commercialization of DPSC and DPSC secretome-based therapeutics for dental pulp regeneration requires overcoming market-related and translational bottlenecks. One major hurdle is establishing scalable manufacturing processes that preserve the biological efficacy of DPSCs and their secretome while meeting regulatory expectations for reproducibility and safety^162,163^. Despite promising preclinical and early clinical data, the absence of long-term efficacy and safety outcomes continues to delay investor confidence and broader market adoption^77^. Additionally, pricing and reimbursement strategies for regenerative dental therapies remain unclear, as payers and regulatory agencies may struggle to classify and appraise the cost-effectiveness of such biologics, especially cell-free therapies like the secretome. Intellectual property (IP) protection presents another challenge due to the difficulty in patenting naturally derived secretome products unless innovative processing or formulation methods are involved^163^. Furthermore, there is limited standardization in clinical protocols and outcome metrics, which complicates cross-study comparison and real-world evidence generation. Addressing these issues through international collaboration, harmonized regulatory guidance and robust clinical trials could accelerate the integration of DPSC-derived products into mainstream regenerative dentistry and expand their commercial potential globally^159^.

## Conclusion and future direction

Using dental pulp stem cells (DPSCs) and their secretome in conjunction with three-dimensional (3D) scaffolds is emerging as an effective method in regenerative endodontics. DPSCs are an excellent cell source for tissue engineering of pulp tissue since they are highly proliferative, possess multi-lineage differentiation potency and have immunomodulatory properties. DPSC secretome offers a promising, cell-free approach to regenerate the pulp–dentin complex. Growth factors, cytokines and extracellular vesicles are among the many bioactive compounds found in the DPSC-derived secretome that promote angiogenesis, neurogenesis and cell recruitment, thereby aiding in tissue repair and regeneration. When DPSCs and the secretome are combined with 3D scaffolds, they produce a biomimetic microenvironment that mimics natural dental pulp, which promotes advantageous cell adhesion, proliferation and differentiation. In order to restore the pulp–dentin complex, the 3D scaffold can also impart advantageous physical and chemical properties like mechanical strength, biodegradability and porosity that promote dentin–pulp regeneration^137^. By offering biologically based techniques for pulp regeneration instead of inert materials, this synergistic approach has the potential to drastically alter endodontic treatment. The next steps in research should concentrate on scaffold design and functionalization optimization, DPSC secretome production standardization, and thorough *in vivo* experiments to confirm the clinical translation of these approaches. With continued progress in these areas, the combination of DPSCs and their secretome with 3D scaffolds may be the paradigm shift that regenerative dentistry needs to assume its rightful place in clinical practice. Although the combination of DPSCs and their secretome with 3D scaffolds is undoubtedly an exciting development in regenerative dentistry, further work is necessary to overcome challenges that could impede its further development and full clinical reach. Future work should focus on scaffold design optimization to better mimic the native extracellular matrix, if possible, by incorporating dynamic, bio-responsive properties to be able to mimic changes in the pulp microenvironment. Further work is also necessary to scale up and standardize DPSC secretome production, while continuing to identify and isolate the important bioactive components in the secretome. To ensure consistency and reproducibility in the biomaterial production, these efforts will assist in addressing these challenges in regenerative dentistry. In addition, advanced bioprinting methods could be used to create scaffolds that are customized for patients and can include specific architecture and functionality. Thorough *in vivo* studies and preclinical investigations will be necessary to evaluate the safety, effectiveness and long-term effects of these regenerative strategies. Bioinformatics and computational analysis can enrich the understanding of the cellular context within the scaffold, which will accelerate therapeutic evolution and refinement. It will also be important to tackle the regulatory and translational challenges and facilitate bringing the biomaterial from the bench to the clinic, which will support expanding the incorporation of this technological approach into clinical practice for regenerative endodontics.

## Data Availability

No new data were generated or analyzed in this study. All references used are publicly available and properly cited.
